# Public Continuum Beliefs for Different Levels of Depression Severity

**DOI:** 10.3389/fpsyt.2021.666489

**Published:** 2021-06-09

**Authors:** Anna C. Makowski, Georg Schomerus, Olaf von dem Knesebeck

**Affiliations:** ^1^Department of Medical Sociology, University Medical Center Hamburg-Eppendorf, Hamburg, Germany; ^2^Department of Psychiatry and Psychotherapy, University Hospital Leipzig, Leipzig, Germany

**Keywords:** continuum beliefs, depression, symptom severity, population survey, Germany

## Abstract

**Introduction:** The notion that depression is a disorder that moves along a continuum is well-established. Similarly, the belief in the continuity of mental illness is considered an important element in the stigma process. Against this background, it is the aim of this study to examine whether public continuum beliefs vary with the severity of depressive symptoms.

**Methods:** Analyses were based on computer-assisted telephone interviews (CATIs) conducted in winter 2019/2020 in Germany (*N* = 1,009, response rate 46.8%). Using three vignettes representing mild, moderate, and severe depressive symptoms, beliefs regarding the continuity of symptoms, specifically a fundamental difference, were assessed with seven items. Sociodemographic characteristics and own experiences with depression (affliction and contact) were introduced as covariates.

**Results:** Significant differences between the three groups of severity were found for the majority of the items measuring continuum beliefs or perceived fundamental difference. However, only few items showed a linear trend indicating a parallel between symptom severity and beliefs. Multivariate regression models showed that a moderate degree of depression was positively associated with stronger continuum beliefs but also with greater perceived difference compared to the mild degree, while no significant associations emerged for the severe vignette.

**Limitations:** Although a comparison of our sample with official statistics supports the external validity, we cannot rule out a selection bias. It is arguable in how far short case vignettes convey a holistic picture of a person affected by depressive symptoms.

**Conclusion:** Our results do not indicate a parallel between symptom severity and public continuum beliefs.

## Introduction

In recent years, also surrounding the revision of classification systems such as the International Classification of Diseases [ICD-10 (1)] or Diagnostic and Statistical Manual of Mental Disorders [DSM, (2)], research has increasingly focused on a dimensional vs. a categorical approach toward mental health and illness (3, 4). Regarding affective disorders, a literature review concluded that depressive disorders are rather conceptualized along a continuum with increasing symptom severity (5), and Bowins (6) contended that depression as a continuum is best characterized by duration and severity dimensions. The presence of depressive symptoms among non-depressed individuals has also been shown in epidemiological studies. The symptom of sadness has been found to be prevalent among 17.8% of respondents without major depressive disorder (MDD), and it could be shown that depressive symptoms ranged from non-pathological sadness to MDD according to DSM-V (7). These findings could be replicated in a second study with a representative US sample (8).

Concerning stigma research, the concept of continuity of mental health and illness vs. a dichotomy also plays an important role. This becomes evident when looking at the different steps of the stigmatization process as described by Link and Phelan (9). They have conceptualized stigma as the convergence of the following components: (1) distinguishing and labeling human differences that are socially relevant, (2) linking these labeled differences to undesirable characteristics (negative stereotypes), (3) separating “us” from “them” on the basis of these social labels, and lastly, (4) labels and separation leading to discrimination and status loss (9). A central aspect of this model is the separation of and clear boundary between “us” and “them,” and people with mental illness may be regarded as fundamentally different and stigmatized against (10). Perceived otherness, or rather its opposite, similarity and the belief in the continuity of mental illness, has been taken up by researchers in the field of mental illness stigma.

In the first study on continuum beliefs, Schomerus et al. (11) found that 42% of the respondents agreed to a continuity of depressive symptoms, 27% to a symptom continuity in schizophrenia, and 26% in alcohol dependence. Furthermore, continuum beliefs were associated with more positive emotional reactions and less desire for social distance. In the subsequent years, national and international studies came to similar results (12–17). This has led to an increased focus on the dimensionality of mental health and illness in approaches that aim to reduce the stigma toward persons with psychiatric disorders. Continuum messages had positive effects on the view that persons with mental illness are “different” as well as on recovery beliefs when compared to neutral or categorical messages (18). Further studies could also show that strengthening continuum beliefs has the potential to reduce public mental illness stigma (19, 20).

Many studies on the public stigma of mental illness make use of vignettes, in which a patient with typical signs and symptoms of a psychiatric disorder is described (11, 14, 21). This is then followed by questions regarding, for example, attitudes toward this person, including continuum beliefs. These vignettes usually do not vary in the severity of symptoms. Rather, studies use one vignette presenting an afflicted person with moderate to severe symptoms. Thus, there is not much known about variations in public stigma in general and in continuum beliefs in particular according to the severity of the symptoms presented.

Against this background, it is the aim of this study to examine whether public continuum beliefs vary with the severity of depression symptoms. Moreover, associations between symptom severity, sociodemographic characteristics, experience with depressive symptoms, and continuum beliefs will be analyzed.

## Materials and Methods

### Study Design and Sample

The analyses are based on representative data from computer-assisted telephone interviews (CATIs) that were conducted between November 2019 and January 2020 in Germany. Sampling included registered as well as non-registered telephone numbers via random digital dialing. As the share of German adults solely relying on their mobile phone was 14% in 2018 (22), we chose to use a dual-frame approach. This incorporated a share of 30% of mobile numbers in the gross sample, ensuring to reach those mobile-only users and otherwise hard-to-reach target groups.

Regarding mobile users, the target person was the one answering the phone. In case this was someone younger than 18 years of age, the connection was considered a neutral dropout. For landline numbers, the Kish-selection grid (23) was applied to randomly select a person from the household.

The overall sample in this study consisted of *N* = 1,009 adult participants (≥18 years of age). To obtain this sample size, *N* = 2,145 persons were randomly contacted. Of those, *n* = 625 were not available, and *n* = 520 chose not to participate. This led to a response rate of 46.8%.

The study was approved by the Local Psychological Ethics Committee at the Center for Psychosocial Medicine, University Medical Center Hamburg (No. LPEK-0091). As the interviews were conducted by telephone, respondents were verbally informed about the study and asked to participate. Their consent or refusal was documented by the interviewer.

Regarding the distribution of sociodemographic characteristics, comparisons with official statistics have shown that our sample is similar to the general German adult population in terms of sex, age, and level of education (also see Table 1).

**Table 1:** Sociodemographic variables of the sample compared to official statistics (in %).

	**Sample (*n* = 1,009)**	**Official statistics**	***p*(χ^2^)**
**Gender**			
Female	51.1	50.7^a^	0.486
**Age groups**			
18– ≤ 24 years	6.8	9.1^b^	0.518
25– ≤ 39 years	13.4	21.2^b^	
40– ≤ 59 years	35.8	33.8^b^	
60– ≤ 64 years	12.6	10.1^b^	
≥65 years	31.4	25.8^b^	
**Level of education**			
≤ 9 years	35.9	35.7^c^	0.050
10 years	30.6	30.9^c^	
≥12 years	33.5	33.1^c^	

a *Federal Office of Statistics (Destatis) 2019 p. 26;*

b *Federal Office of Statistics (Destatis) 2019 p. 31;*

c *Federal Office of Statistics (Destatis) 2018 p. 21; weighted data*.

### Vignettes

This study made use of a vignette design to elicit knowledge and attitudes on depression and persons afflicted. There were three unlabeled vignettes each representing a different degree of depression severity: mild (*n* = 353), moderate (*n* = 334), and severe depression (*n* = 322). For the development of the different case stories, psychiatrists and clinical psychologists were consulted. The different signs and symptoms for the respective depressive symptomatology were based upon the International Classification of Diseases (ICD), 10^th^ edition (1) and the National Clinical Care Guideline for Depression (24). Here, the different degrees of depression severity are classified according to the number as well the severity of symptoms presented (please see Appendix for vignettes). The specific vignettes for the different severity levels were designed in multiple consultations with clinical experts. The clinicians emphasized the best possible operationalization and a clear differentiation of symptom severity based on ICD 10. At the same time, the design of the case histories had to take into account the requirements of the telephone survey (comprehensibility, length of the vignette). A trained speaker audio-recorded the three vignettes, which were then played to the respondents directly from the computer in order to neutralize interviewer-associated effects. The sex of the fictive patients in the vignettes was systematically varied.

### Measures

To assess the respondents' agreement and disagreement with a continuum of depressive symptomatology, we made use of items introduced by Schomerus et al. (13). With seven items, it was enquired in how far the problems of the person in the vignette (Mrs. D. or Mr. D.) were considered as something on a continuum or were perceived as fundamentally different (please see Table 2). In contrast to the instrument by Schomerus et al. (13), a four-point Likert scale was chosen in the present study (from 1 “totally disagree” to 4 “totally agree”). The elimination of the neutral middle category served to align the answer options with other questions in the survey. Respondents who could not or did not want to make a decision when answering the questions were given the option of choosing residual categories (“don't know” and “prefer not to say”).

**Table 2:** Agreement (in %) to single items of the continuum of depression and mean values and [standard deviation (SD)] for subscales (χ^2^-test for categorical, *F*-test for mean differences, data weighted, *N* = 1,009).

**Single items and corresponding subscale**	**Level of agreement in % with [95% CI]** ***Mean values (SD)*** **for subscales**			***p*** **χ^2^ or**
				***ANOVA***
	**Mild** **(*n* = 353)**	**Moderate** **(*n* = 334)**	**Severe** **(*n* = 322)**	
There is something about Mrs./Mr. D. that makes her fundamentally different from other people.	56.2 [51.0–61.4]	65.9 [60.7–71.1]	67.4 [62.2–72.6]	**0.004**
Someone with arthritis or a broken leg has just one thing wrong with them, but a person like Mrs. /Mr. D. is fundamentally different from other people.	48.8 [43.5–54.1]	46.7 [41.3–52.1]	49.3 [43.7–54.9]	0.770
Mrs. /Mr. D. is in a state of mind that normal people simply cannot understand.	59.1 [53.9–64.3]	66.4 [61.3–71.5]	68.4 [63.3–73.5]	**0.028**
Overall, Mrs. /Mr. D.'s problems are abnormal.	38.3 [33.2–43.5]	45.5 [40.1–50.9]	36.1 [30.8–41.2]	**0.036**
*Subscale “Perceived Fundamental Difference”*.	*2.49 (0.66)*	*2.59 (0.70)*	*2.56 (0.67)*	*F*_2, 996_ = *2.06* *p* = *0.128*
Sometimes we are all at least a little like Mrs. /Mr. D.; it is only the question how pronounced this state is.	79.4 [75.1–83.7]	87.0 [83.4–90.6]	79.7 [75.3–84.1]	**0.015**
To some extent, most persons will experience problems that are similar to those of Mrs. /Mr. D.	79.5 [75.3–83.7]	74.8 [70.1–79.5]	66.8 [61.6–72.0]	**0.001**
People with problems like Mrs. /Mr. D. are normal persons like everybody else.	86.3 [82.7–89.9]	87.1 [83.5–90.7]	80.8 [76.5–85.1]	**0.049**
*Subscale “Continuum Belief”*.	*3.09 (0.62)*	*3.20 (0.55)*	*3.09 (0.72)*	*F*_2, 1004_ = *3.13* *p* = ***0.044***

*Statistically significant values (p < 0.05) are in bold*.

Respondents were also asked regarding their personal experience with the symptomatology presented in the vignette. The interviewers asked whether the respondent is or has been affected by such a symptomatology himself or herself. Additionally, they asked whether the interviewees had or have had personal contact with people with such complaints. The possible answers to these questions were “yes,” “no,” and “prefer not so say.” Furthermore, sociodemographic data of the participants were collected at the end of the interview. Of these, sex, age, and education were included in the analyses.

### Statistical Analyses

The descriptive analyses will be presented as proportions (%) or means (with standard deviation, SD). For a condensed and descriptive presentation of the results, the items measuring agreement with either a continuum or fundamental difference of depressive symptomatology were dichotomized (totally disagree/disagree vs. totally agree/agree). χ^2^-tests [3 ×2 tables] were applied to test for differences in levels of agreement between the three groups (mild, moderate, or severe depression vignette).

In order to achieve a dimension reduction and increase the interpretability of data on continuum belief/fundamental difference, the seven items measuring (dis-) agreement with continuum beliefs were entered into a principal component analysis (PCA), following Schomerus et al. (13). As the underlying constructs can be assumed to be related, oblique rotation was applied. On the basis of the two extracted components (please see the Results section for details), mean scores were computed. For between-group differences of those mean scores, analyses of variance (ANOVA) were performed. In a next step, the scores were entered into multiple linear regression models to test for associations with a belief in continuity of symptoms, specifically fundamental difference. Severity of symptoms, sex, age, level of education, personal affliction, as well as contact to someone afflicted served as predictors in the models.

All statistical tests were conducted using R (25). For analyses of variance, the function anova was used, the PCA was carried out using the package principal_component (26), while linear regression analyses were carried out with the function lm. For all analyses, detailed *p*-values are reported. Values <0.05 are regarded as statistically significant.

## Results

In Table 1, sociodemographic characteristics of the sample are briefly described. A comparison with official statistics shows that the distribution of gender, age, as well as level of education is similar to that in the general German population (27, 28).

The principal component analysis (PCA) resulted in two components with eigenvalues > 1 (please see Table A in the Appendix). The first component (four items) was termed “Perceived Fundamental Difference” (eigenvalue 1.80, explained variance 25.7%, Cronbach's alpha 0.58, mean inter-item-correlation 0.26). The second component (three items) had an eigenvalue of 1.61 (explained variance 23.0%, Cronbach's alpha 0.57, mean inter-item-correlation 0.30) and was termed “Continuum Belief.” Further details on properties of item and scales can be found in Table B in the Appendix. Mean scores ranging from 1 to 4 were computed for the two components “Perceived Fundamental Difference” and “Continuum Belief.”

In Table 2, levels of agreement (in %) to the single items of belief in a continuum or fundamental difference as well as the mean values of the corresponding subscales are reported. Tests between groups (vignettes) revealed statistically significant differences for most of the items. Regarding the subscale “Perceived Fundamental Difference,” agreement was significantly lower in case of the mild vignette for two items (“Mrs./Mr. D. is fundamentally different, Mrs./Mr. D. is in a state of mind one cannot understand.”). However, the mean values of this subscale did not significantly differ.

With regard to the subscale “Continuum Beliefs,” all three items as well as the subscale itself showed significant differences between the three vignettes. Levels of agreement declined from mild to severe depression for the item that most people will—to some extent—experience similar problems (p=0.001). Regarding one item (“Sometimes we are all at least a little like Mrs./Mr. D …”) as well as the subscale, the moderate vignette showed the highest values of agreement.

Results of the linear regression analyses with the scale “Continuum Belief” as the dependent variable can be seen in Table 3. Personal experience with the symptomatology presented in the vignettes emerged as the strongest predictor. Being afflicted oneself as well as having contact to someone affected was significantly associated with a stronger continuum belief. The degree of severity in the presented vignettes was also of importance, at least partially. Compared to the vignette with mild symptomatology, the moderate depression vignette was significantly related with the greater continuum belief.

**Table 3:** Multiple linear regression, sociodemographic characteristics, personal experience, and level of severity regressed on “Continuum Belief” (*N* = 985).

	**B**	**ß**	**95% CI**	***P***
**Sex (ref. male)**	0.06	0.05	−0.01–0.14	0.108
**Age**	−0.00	−0.02	−0.00–0.00	0.441
**Level of education (ref**. **≤ 9 years)**				
10 years of education	0.04	0.06	−0.06–0.14	0.445
≥ 12 years of education	0.04	0.06	−0.06–0.14	0.443
**Own experience (ref. no)**	0.27	0.21	0.19–0.35	**<0.001**
**Contact to someone with depression (ref. no)**	0.31	0.19	0.21–0.41	**<0.001**
**Level of severity depression in vignette (ref. mild depression)**				
Moderate depression	0.10	0.16	0.01–0.19	**0.028**
Severe depression	0.01	0.02	−0.08–0.11	0.773
R^2^/R^2^ adjusted	0.108/0.101			

*Statistically significant values (p <0.05) are in bold*.

In Table 4, the results of the multiple regression analysis with the scale of “Perceived Fundamental Difference” are reported. The belief of fundamental difference was significantly associated with the higher age of the respondents. Among respondents with a higher level of education (≥12 years vs. ≤ 9 years) and with contact to someone afflicted, belief of fundamental difference was significantly less pronounced, while own experience was positively associated. Finally, perceived fundamental difference was more pronounced among respondents who faced the moderate depression vignette.

**Table 4:** Multiple linear regression, sociodemographic characteristics, personal experience, and level of severity regressed on “Perceived Fundamental Difference” (*N* = 977).

	**B**	**ß**	**95% CI**	***P***
**Sex (ref. male)**	0.03	0.02	−0.06–0.11	0.544
**Age**	0.01	0.23	0.01–0.01	**<0.001**
**Level of education (ref**. **≤ 9 years)**				
10 years of education	−0.00	−0.00	−0.10–0.10	0.989
≥12 years of education	−0.21	−0.31	−0.32−0.11	**<0.001**
**Own experience (ref. no)**	0.09	0.06	0.00–0.17	**0.040**
**Contact to someone with depression (ref. no)**	−0.14	−0.08	−0.24−0.03	**0.011**
**Level of severity depression in vignette (ref. mild depression)**				
Moderate depression	0.13	0.19	0.03–0.23	**0.008**
Severe depression	0.06	0.09	−0.04–0.16	0.224
R^2^/R^2^ adjusted	0.128/0.120			

*Statistically significant values (p <0.05) are in bold*.

## Discussion

The present study focused on public continuum beliefs and on the magnitude of variation according to different severity degrees of depression. The notion that depression is a disorder that moves along a continuum has been pursued for some time. Similarly, perceived otherness, or rather its opposite, similarity and the belief in the continuity of mental illness, is considered an important element in the stigma process (9, 11, 12). However, studies in stigma research generally do not use vignettes with varying severity of symptomatology to collect such attitudes. To the best of our knowledge, this is the first study to test in how far a continuum of symptoms is reflected in varying beliefs of the public.

Using three different vignettes representing mild, moderate, and severe depressive symptoms, the public's beliefs regarding the continuity of symptoms, specifically a fundamental difference, were elicited. Based on Schomerus et al. (13), we used seven items that could be assigned to two subscales in principal component analysis: “Continuum Belief” and “Perceived Fundamental Difference.” In addition to testing differences between the groups (vignettes), we applied regression analyses to investigate associations between symptom severity and the two subscales controlling for several covariates.

Statistically significant differences between the three groups of severity were found for the majority of the items measuring continuum beliefs or perceived differences. However, only few items showed a linear trend indicating a parallel between symptom severity and beliefs about continuity or fundamental difference. Beyond that, differences between the vignettes were inconsistent. In some cases, agreement was lowest for the mild vignette, while for other items, comparably high agreement was observed in the case of the moderate vignette. Furthermore, it is remarkable that, even in the case of rather mild depressive symptoms, there was 38 to 56% agreement concerning items measuring fundamental difference. On the other hand, in case of severe symptoms, a majority (67 to 81%) of the population agreed to statements assessing continuum beliefs.

In terms of the two subscales, differences were not or marginally significant in the descriptive analyses. The subsequent multivariate regression models showed that a moderate degree of depression was positively associated with both subscales (i.e., stronger continuum beliefs and greater perceived difference) compared to the mild degree, while no significant associations emerged for the severe vignette. Thus, in a way, conflicting and ambiguous findings are confirmed when variables were controlled that are known to be associated with different aspects of the stigma process (29–31). Overall, our results do not indicate a parallel between symptom severity and public beliefs about continuity or fundamental difference.

Furthermore, significant positive associations between personal experiences with depression (being affected oneself or contact to someone affected) and the subscale “Continuum Belief” occurred. Similar results were obtained in a study among an Asian community that explored the associations between contact and a single item measuring continuum belief (29), while Buckwitz et al. (30) showed that participants with former positive contact displayed significantly greater continuum beliefs than those without contact. This appears to be quite comprehensible, since one's own involvement or contact with those affected blurs the line between “us” and “them.” It is also known from stigma research that positive contact is associated with positive attitudes and less prejudice toward people affected by mental illness (31), and it has been found to be an effective intervention measure to improve stigma-related attitudes in the short term (32). Moreover, Corrigan et al. (18) were able to show that continuum messages in combination with former interpersonal contact go along with reduced mental illness stigma. In addition, being or having been affected by depression may as well best demonstrate the continuity of depressive symptoms to oneself, which is then reflected in personal continuum beliefs.

Personal experiences also displayed significant associations with the subscale “Perceived Fundamental Difference.” Contact to those affected was associated with less perceived difference, which again speaks for the fact that knowing or having met someone with depression dissolves perceived boundaries between “us” and “them.” However, it is noteworthy that own affliction displayed a positive relationship with fundamental difference. Although weak, this can be interpreted in several ways: as an indication of internalized stigma (33), or reflecting the experience of being regarded as different by others, or referring to personally feeling fundamentally different from other people during a depressive episode. The strongest associations with “Perceived Fundamental Difference” occurred with older age as well as with a lower level of education. There has already been extensive research with regard to age and education in relation to other dimensions of the stigma process, which confirms our results. For example, it is known that higher age as well as lower levels of education are associated with greater desire to socially distance oneself from those affected by mental disorders (29, 30, 34–36).

## Limitations

Our findings need to be evaluated and discussed in the light of some limitations. Although a response rate of 46.8% can be considered adequate for a telephone interview (37), we cannot rule out a selection bias. However, the comparison of sociodemographic variables of our sample with official statistics supports the external validity.

Following Schomerus et al. (13), we have used seven items to determine the continuum belief, specifically the assumption that someone with such symptoms is fundamentally different. The primary goal of the analysis was to provide a reduced solution for easier interpretation, which is why the items were analyzed using PCA (38), analogous to the approach used by Schomerus et al. The results of the PCA performed in this study are only partially comparable to those of the original version. Our factor loadings were distinct, and no cross-loadings were present. However, in contrast to the work of Schomerus et al., one item displayed a positive loading on the component “Perceived Fundamental Difference” that loaded negatively on “Continuum Beliefs” in the original. Furthermore, it must be noted that Cronbach's α of the present subscales can be regarded acceptable at best. Cronbach's α barely reached 0.6, which is deemed an acceptable cutoff value (39). However, the mean inter-item correlation, which is an alternative measure to indicate acceptability, displayed satisfactory values for the subscales with ranges between 0.2 and 0.4 (40). Designs using case vignettes as a stimulus to elicit public attitudes are quite common in the field of mental health research, as they proved useful. The content can be modified to be consistent with the researchers' topics, vignettes can be perceived as relaxing and interesting by participants, and most importantly, by achieving depersonalization, they can facilitate moving beyond personal situations toward generating responses on a social level (41). It is arguable in how far case stories convey a holistic picture of a person who is affected by depressive symptoms. However, the vignettes must not be too long, especially when they are being used in telephone interviews. Time restrictions in the interviews make it necessary that the vignette content is understood as best as possible by the participants at the beginning of the interview. Simultaneously, the case stories must be formulated so clearly and briefly that they can be recalled in the course of the interview. Furthermore, with regard to our research question, it would have been optimal if the severity of depression could also have been measured in terms of continuous vignettes to further account for the continuum of depression. Unfortunately, this was simply not feasible in our planned design. The vignettes were developed together with experts in the field and are based on national and international guidelines. These define that the severity of depression is determined by the number of symptoms, among other things, that is, the more symptoms are present, the more severe the disorder. During development, the clinical experts took care to ensure that the three case histories could be clearly distinguished from each other while being comprehensible to laypersons. Therefore, there are slight differences between the vignettes regarding their length. The number of symptoms may have had an impact on the extent to which participants were able to develop an attitude regarding continuum beliefs or fundamental difference.

## Conclusion

The notion that depressive symptoms move along a continuum has become quite established, and also in stigma research, the continuum idea plays an important role. Overall, we did not find a parallel between symptom severity and public continuum beliefs. Our results, in a way, imply a positive and a negative message: on the one hand, the large majority of the public believes in a continuity of depression for all three levels of symptom severity. On the other hand, there is a clear evidence for a separation between “us” and “them” even for mild stages of depression. Previous studies have shown that strengthening continuum beliefs has the potential to reduce public mental illness stigma (19, 20). In this regard, results indicate that such interventions may also be advisable for mild forms of depression.

## Data Availability Statement

The raw data will be made available by the corresponding author upon reasonable request.

## Ethics Statement

The studies involving human participants were reviewed and approved by Local Psychological Ethics Committee at the Center for Psychosocial Medicine, University Medical Center Hamburg. Written informed consent for participation was not required for this study in accordance with the national legislation and the institutional requirements. Respondents were verbally informed about the study and asked for consent. Participants' consent or refusal was documented.

## Author Contributions

OK and AM planned the study and interpreted results. GS was involved in the design of vignettes and questionnaire. AM carried out the statistical analyses and wrote the first draft of the manuscript. OK and GS took part in drafting the manuscript and critically revised it. All authors approved the final version of the manuscript.

## Conflict of Interest

The authors declare that the research was conducted in the absence of any commercial or financial relationships that could be construed as a potential conflict of interest.
