# Supplementary material for: Public Continuum Beliefs for Different Levels of Depression Severity
Source: Front Psychiatry. 2021 Jun 9;12:666489. doi: 10.3389/fpsyt.2021.666489 (PMC8219923; doi:10.3389/fpsyt.2021.666489)
Supplement: Supplementary file 1 [file Data_Sheet_1.docx]

# Appendix

Vignettes

Minor depression

*37-year-old Denise D. ^*^ has been feeling down on and off for the past few months. Mrs. D. has little interest in everyday things.*

*She sometimes finds it difficult to fall asleep, and at work Mrs. D. can no longer concentrate very well. In everyday life Mrs. D. is impaired, but she can cope with most activities.*

Moderate depression

*37-year-old Denise D. ^*^ has often felt so down for the past few months that nothing can cheer her up. Mrs. D. has lost interest in everyday things.*

*She often has trouble falling asleep in the evenings and often feels tired and weak in the mornings. At work Mrs. D. has difficulty concentrating. Mrs. D. feels that she is not good enough. Overall, Mrs. D. has great difficulty coping with her everyday life.*

Severe depression

*For several months now, 37-year-old Denise D. ^*^ has been feeling so down all the time that nothing can cheer her up. Mrs. D. no longer has anything that she enjoys or is happy about.*

*It takes her a long time to fall asleep in the evenings and she wakes up frequently at night. In the mornings she is always tired and powerless. If she manages to go to work, she can no longer concentrate at all. Mrs. D. has the feeling that she is not good enough and she doubts that her life still makes any sense at all.*

*^*^*Gender in the vignettes was systematically varied.

Results of Principal Component Analysis and item statistics

Table A Component loadings and Keyser-Meyer-Olkin Measure of sampling adequacy (MSA) (N=1,009)

|  | Component 1  'Perceived Fundamental Difference' | Component 2 'Continuum Belief' | MSA |
| --- | --- | --- | --- |
| *There is something about Mrs./Mr. D. that makes her fundamentally different from other people.* | **0.72** | 0.02 | 0.66 |
| *Someone with arthritis or a broken leg has just one thing wrong with them, but a person like Mrs./Mr. D. is fundamentally different from other people.* | **0.69** | -0.11 | 0.68 |
| *Mrs./Mr. D. is in a state of mind that normal people simply cannot understand.* | **0.66** | 0.13 | 0.69 |
| *Overall, Mrs./Mr. D.’s problems are abnormal.* | **0.60** | -0.04 | 0.72 |
| *Sometimes we are all at least a little like Mrs./Mr. D., it is only the question how pronounced this state is.* | 0.06 | **0.77** | 0.58 |
| *To some extent, most persons will experience problems that are similar to those of Mrs./Mr. D.* | 0.00 | **0.76** | 0.59 |
| *People with problems like Mrs./Mr. D. are normal persons like everybody else.* | -0.09 | **0.64** | 0.66 |

Table B Properties of item scales (N=1,009)

|  | Missing (%) | Skew | Item difficulty | Item discrim-nation | Mean inter item correlation | Cronbach’s α |
| --- | --- | --- | --- | --- | --- | --- |
| *There is something about Mrs./Mr. D. that makes her fundamentally different from other people.* | 1.78 | -0.36 | 0.69 | 0.40 | 0.26 | 0.58 |
| *Someone with arthritis or a broken leg has just one thing wrong with them, but a person like Mrs./Mr. D. is fundamentally different from other people.* | 1.98 | 0.07 | 0.60 | 0.38 |  |  |
| *Mrs./Mr. D. is in a state of mind that normal people simply cannot understand.* | 1.49 | -0.38 | 0.69 | 0.36 |  |  |
| *Overall, Mrs./Mr. D.’s problems are abnormal.* | 1.59 | 0.29 | 0.56 | 0.32 |  |  |
| *Sometimes we are all at least a little like Mrs./Mr. D., it is only the question how pronounced this state is.* | 1.19 | -0.88 | 0.77 | 0.42 | 0.30 | 0.57 |
| *To some extent, most persons will experience problems that are similar to those of Mrs./Mr. D.* | 0.89 | -0.45 | 0.72 | 0.41 |  |  |
| *People with problems like Mrs./Mr. D. are normal persons like everybody else.* | 0.30 | -1.47 | 0.86 | 0.30 |  |  |
